# Antifungal treatment of wild amphibian populations caused a transient reduction in the prevalence of the fungal pathogen, *Batrachochytrium dendrobatidis*

**DOI:** 10.1038/s41598-017-05798-9

**Published:** 2017-07-20

**Authors:** Corina C. Geiger, Cindy Bregnard, Elodie Maluenda, Maarten J. Voordouw, Benedikt R. Schmidt

**Affiliations:** 1 Department of Evolutionary Biology and Environmental Studies, University of Zurich, Zurich, Switzerland; 2 Laboratory of Ecology and Evolution of Parasites, Institute of Biology, University of Neuchâtel, Neuchâtel, Switzerland; 3 KARCH, Neuchâtel, Switzerland

## Abstract

Emerging infectious diseases can drive host populations to extinction and are a major driver of biodiversity loss. Controlling diseases and mitigating their impacts is therefore a priority for conservation science and practice. Chytridiomycosis is a devastating disease of amphibians that is caused by the fungal pathogen *Batrachochytrium dendrobatidis* (*Bd*), and for which there is an urgent need to develop mitigation methods. We treated tadpoles of the common midwife toad (*Alytes obstetricans*) with antifungal agents using a capture-treat-release approach in the field. Antifungal treatment during the spring reduced the prevalence of *Bd* in the cohort of tadpoles that had overwintered and reduced transmission of *Bd* from this cohort to the uninfected young-of-the-year cohort. Unfortunately, the mitigation was only transient, and the antifungal treatment was unable to prevent the rapid spread of *Bd* through the young-of-the year cohort. During the winter, *Bd* prevalence reached 100% in both the control and treated ponds. In the following spring, no effects of treatment were detectable anymore. We conclude that the sporadic application of antifungal agents in the present study was not sufficient for the long-term and large-scale control of *Bd* in this amphibian system.

## Introduction

Emerging infectious diseases have been recognized as important drivers of the loss of biodiversity^1^. An increasing number of emerging fungal diseases is causing host mass mortality, extirpating local populations, and threatening biodiversity^2^. These diseases can cause population declines because all host individuals can become infected before host population size falls below a threshold that would halt an epizootic^2^. Chytridiomycosis is an emerging infectious fungal disease of amphibians which has become a model system for the study of disease-induced host population decline and extinction^3^. The etiological agent of chytridiomycosis is the chytrid fungus *Batrachochytrium dendrobatidis* (*Bd*) (Chytridiomycota), which can infect larval, metamorph, and adult amphibians^4^ (a second pathogenic chytrid fungus was recently described^5^). *Bd* infects a wide range of amphibian species, some acting as a reservoir for the pathogen^6^. Even though *Bd* is responsible for amphibian mass mortality, population extirpations, global extinctions of species, and the loss of phylogenetic diversity^7, 8^, there is no established method to mitigate the effects of the disease in wild populations^9–12^. However, it is known from agriculture that fungal pathogens of plants can be controlled using fungicides. Fungicides are known to reduce *Bd* infection^13^ and two proof-of-concept studies have shown that fungicides can be used to control or even eradicate *Bd* in the wild^14, 15^.

The efficacy of mitigation depends on the *Bd* infection prevalence of the different host life history stages and the sensitivity of the host population growth rate to stage-specific disease-induced mortality^16^. *Bd* does not seem to affect the survival of tadpoles, which allows this stage to act as an intraspecific reservoir for *Bd* ^17–19^. In contrast, *Bd* reduces survival of post-metamorphic individuals^20, 21^, the life history stage to which the amphibian population growth rate is highly sensitive^22–24^. Fungal treatments that clear the infection or that reduce the infection load in tadpoles before metamorphosis may therefore reduce mortality in post-metamorphic juveniles and increase population viability^16^. Such treatments may further reduce disease prevalence in amphibian species where *Bd* transmission occurs between overlapping generations of tadpoles.

The common midwife toad (*Alytes obstetricans*) is a good model system for studying the epidemiology and control of *Bd* because this host is highly susceptible and commonly infected with this pathogen^21, 25, 26^. This species has suffered *Bd*-driven population declines and extirpations and is red-listed in some European countries^27–29^. The tadpoles of this species overwinter so that in the summer there are often multiple cohorts of tadpoles present in the same pond. After overwintering, *Bd* prevalence is often very high, which may lead to pathogen transmission to young-of-the-year tadpoles^21, 26, 30^. In a previous laboratory study, we had shown that treatment with an antifungal agent (General Tonic) could reduce *Bd* prevalence and load in *A. obstetricans* tadpoles^31^. The purpose of the present study was to use antifungal agents to reduce the prevalence and infection intensity (zoospore load) of *Bd* in natural populations of the common midwife toad. We predicted that antifungal treatment of tadpoles would reduce *Bd* prevalence and load, allow tadpoles to metamorphose into a disease-free state, and reduce *Bd* transmission between overlapping cohorts of tadpoles.

## Results

### Definition of the 2010 and 2011 cohorts

A total of 2096 *Alytes obstetricans* tadpoles were used in the statistical analysis. Tadpoles were classified as belonging to either the overwintering cohort (n = 612) or the young-of-the-year cohort (n = 1484), hereafter referred to as the 2010 and 2011 cohort, respectively. The tadpoles in the 2010 cohort were captured in the months of May, June, and July 2011, whereas the tadpoles in the 2011 cohort were captured in the months of August 2011 to April 2012. The 2010 and 2011 cohorts were analysed separately because they occurred in the pond at different times of the year.

### The *Bd* prevalence in the tadpoles changes over the seasons

A tadpole was classified as infected with *Bd* if it had a *Bd* zoospore load >0.01. The *Bd* prevalence refers to the proportion of tadpoles that were infected with *Bd*. In the statistical analysis of micro-parasite infections, it is standard practice to separately analyse pathogen prevalence and pathogen load (or pathogen burden). The analysis of *Bd* prevalence was based on all of the tadpoles in the data set (n = 2096 tadpoles), whereas the analysis of *Bd* zoospore load (see below) was based on the subset of tadpoles that were classified as being infected with *Bd*.

**Figure 1:**
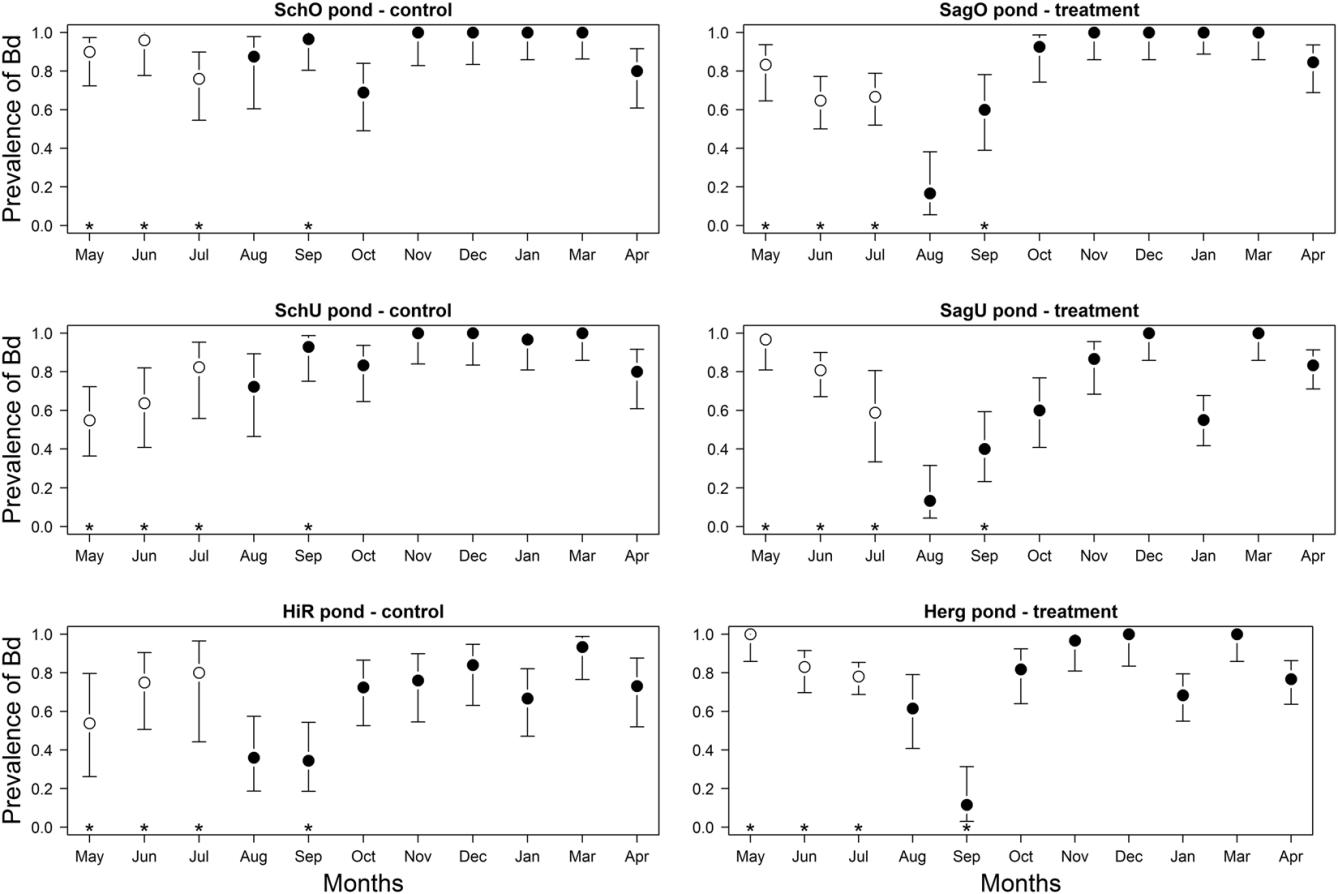
The mean proportion of *Bd*-infected *A*. *obstetricans* tadpoles is shown for each of the six ponds and the eleven months in the study. The control ponds and the antifungal-treated ponds are in the left and right columns, respectively. The 2010 and 2011 cohorts are shown with white and solid black circles, respectively. Fungicide treatment was applied to the tadpoles in the months of May, June, July, and September (asterisks). The bars show the 95% confidence limits of the mean.

The prevalence of *Bd* in the tadpoles was lowest in August and September and highest in the winter in most ponds (Fig. 1). In the 2010 cohort, the *Bd* prevalence remained stable or decreased from May to July, whereas in the 2011 cohort, the *Bd* prevalence increased from August to the winter and then declined in the spring (Fig. 1). In the three treatment ponds (SagO, SagU, Herg), the visible implant elastomer (VIE) tags indicated the number of times that an individual tadpole had been treated with fungicide. In Fig. 1, however, the mean *Bd* prevalence in the three treatment ponds is based on all the tadpoles that were captured, including those that had never been treated with fungicide (i.e. tadpoles with no VIE tags at the time of capture). Similarly, for most of the statistical analyses below, the treatment status of a tadpole (fungicide versus control) depends upon its pond of origin and not whether that particular individual was treated with fungicide or not. For this reason, these statistical analyses are labelled as occurring at the pond level. This approach is justified if fungicide treatment of a fraction of the tadpole population in the treatment ponds reduces the risk of *Bd* transmission to the untreated tadpoles (i.e. herd immunity).

### Effect of the fungicide treatment on the *Bd* prevalence in tadpoles at the pond level

We used generalized linear mixed effects models with binomial errors to model the prevalence of *Bd* as a function of the fungicide treatment (control, treated), the covariate time, and their interaction. Here all tadpoles belonging to the same pond were assigned the same fungicide treatment status (i.e. statistical analysis was done at the pond level). The 2010 and 2011 cohorts were analysed separately because they occurred at different times of the year. The interpretation of the parameter estimates is as follows: the intercept refers to the prevalence of *Bd* in the control ponds on the intercept date (see below for the definition of the intercept date). The fungicide treatment is the difference in *Bd* prevalence between the treated ponds and the control ponds on the intercept date. The covariate time is a slope that indicates whether the prevalence of *Bd* increases or decreases over time. A significant interaction between fungicide treatment and time indicates that the difference in *Bd* prevalence between the control ponds and treated ponds changes over time. In the case of a significant fungicide treatment:time interaction, it is not possible to make a general statement about the effect of the fungicide treatment.

### Definition of the intercept date

In the original analyses, the intercept date was the first date that each cohort was captured in significant numbers, which was May 5, 2011 for the 2010 cohort and August 2, 2011 for the 2011 cohort. For the 2011 cohort of tadpoles (but not the 2010 cohort), it became necessary to include a quadratic effect of time (covariate time^2^) to account for the curvature of the relationship between *Bd* prevalence and time (Fig. 1). However, the strong collinearity between time and time^2^ caused problems with parameter estimation and model convergence. To solve this collinearity problem, the two variables were transformed so that they were orthogonal to each other. One consequence of this rescaling was that the intercept for the 2011 cohort was re-defined as December 15, 2011 (see statistical methods for details and Table S1). Thus for the 2011 cohort, the parameter estimate of the fungicide treatment represents the difference in *Bd* prevalence between the treated ponds and the control ponds on December 15, 2011.

### Effect of the fungicide treatment on the *Bd* prevalence in the 2010 cohort of tadpoles at the pond level

For the 2010 cohort of tadpoles, there was strong support for the interaction between the fungicide treatment and time (support = 95.0%; Tables 1 and S2). In the control ponds, the 95% confidence intervals (CI) of the model-averaged slope of the effect of time included zero (slope = 4.97; 95% CI = −3.76 to 13.70; Table S3) indicating that the prevalence of *Bd* was stable over time (Fig. 1). The 95% CI of the contrast in slopes between the two types of ponds was negative (contrast in slope = −19.52; 95% CI = −30.66 to −8.37; Table S3) indicating that the prevalence of *Bd* declined over time in the treated ponds (Fig. 1). The 95% CI of the difference between the treated and control ponds was positive (contrast in Y-intercept = 1.15; 95% CI = 0.56 to 1.74; Table S3) indicating that on May 5, 2011, prior to any application of the fungicide treatment to the 2010 cohort, the prevalence of *Bd* was higher in the treated ponds than the control ponds.

**Table 1:** Model selection results are shown for the *Bd* prevalence of the 2010 cohort of *A. obstetricans* tadpoles.

ID	F	T	F:T	Random	Df	logLik	AIC	Δ AIC	Weight 1	Weight 2
1	+	+	+	p	5	−313.4	636.8	0.00	0.622	0.62
2	+	+	+	p + m	6	−313.4	638.9	2.04	0.224	0.84
3	+	+	+	p + m + p:m	7	−313.0	640.3	3.44	0.111	0.95

The *Bd* prevalence was modelled as a generalized linear mixed effects model with binomial errors. Of the 20 models in the set, only the top 3 models are shown for which the cumulative support (Weight 2﻿) ≥95.0%. Shown for each model are: the model ID (ID), the fixed effects structure (F = fungicide treatment, T = time, and F:T = interaction), the random effects structure (Random; p = pond identity, m = month identity, p:m = interaction), the model degrees of freedom (Df), the log-likelihood (logLik), the Akaike information criterion (AIC), the difference in the AIC value from the top model (Δ AIC), the model weight (Weight 1), and the cumulative weight (Weight 2). Table S2 shows the results from the full model selection.

### Effect of the fungicide treatment on the *Bd* prevalence in the 2011 cohort of tadpoles at the pond level

For the 2011 cohort of tadpoles, there was moderate support for the interaction between the fungicide treatment and time (support = 48.9%; Tables 2 and S4). The difference between the treated and control ponds was not different from zero (Table S5) indicating that on December 15, 2011, the *Bd* prevalence was the same in the treated and control ponds. In the control ponds, the model-averaged slope of the effect of time was not different from zero (slope = 29.76; 95% CI = −4.17 to 63.70; Table S5) indicating that the prevalence of *Bd* was stable over time (Fig. 1). The 95% CI of the contrast in slopes between the two types of ponds was positive (contrast in slope = 25.57; 95% CI = 2.76 to 48.38; Table S5) indicating that the prevalence of *Bd* increased over time in the treated ponds (Fig. 1). There was also support for a positive quadratic effect of time (support = 68.7% in Tables 2 and S4; slope = 21.68; 95% CI = 2.63 to 60.50), which indicates that *Bd* prevalence was higher in the winter compared to the previous summer or the subsequent spring. In summary, at the start of the 2011 cohort (August 2, 2011), the *Bd* prevalence was lower in the treatment ponds (compared to the control ponds) and therefore it had to increase rapidly over the fall to reach the same high level (~100% prevalence) in the winter (Fig. 1).

**Table 2:** Model selection results are shown for the *Bd* prevalence of the 2011 cohort of *A. obstetricans* tadpoles.

ID	F	T	T^2^	F:T	F:T^2^	Random	Df	logLik	AIC	Δ AIC	Weight 1	Weight 2
32	+	+	+	+		p + m + p:m	8	−575.9	1167.9	0.00	0.237	0.24
16		+	+			p + m + p:m	6	−578.0	1168.1	0.16	0.219	0.46
36	+	+	+	+	+	p + m + p:m	9	−575.5	1169.1	1.16	0.132	0.59
24	+	+		+		p + m + p:m	7	−577.6	1169.3	1.36	0.120	0.71
28	+	+	+			p + m + p:m	7	−577.8	1169.7	1.74	0.099	0.81
12		+				p + m + p:m	5	−579.9	1169.8	1.90	0.092	0.90
20	+	+				p + m + p:m	6	−579.7	1171.4	3.47	0.042	0.94
04						p + m + p:m	4	−581.7	1171.4	3.53	0.041	0.98

The *Bd* prevalence was modelled as a generalized linear mixed effects model with binomial errors. Of the 36 models in the set, only the top 8 models are shown for which the cumulative support (Weight 2) ≥95.0%. Shown for each model are: the model ID (ID), the fixed effects structure (F = fungicide treatment, T = time, T^2^ = quadratic effect of time, F:T = interaction, and F:T^2^), the random effects structure Random; p = pond identity, m = month identity, p:m = interaction), the model degrees of freedom (Df), the log-likelihood (logLik), the Akaike information criterion (AIC), the difference in the AIC value from the top model (Δ AIC), the model weight (Weight 1), and the cumulative weight (Weight 2). Table S4 shows the results from the full model selection.

### The *Bd* zoospore load in the tadpoles changes over the seasons

**Figure 2:**
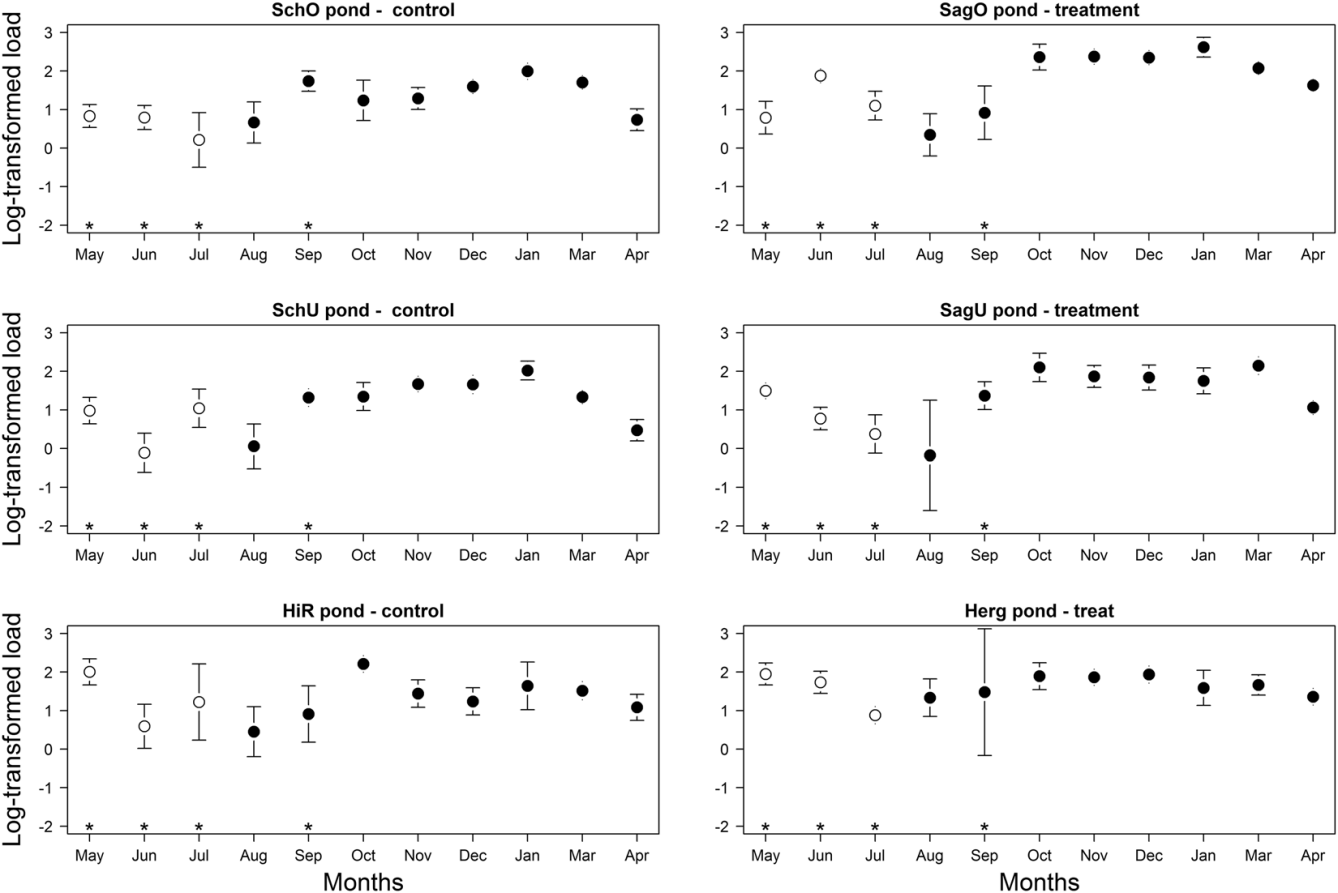
The mean log10-transformed *Bd* zoospore load of the *Bd*-infected *A. obstetricans* tadpoles is shown for each of the six ponds and the eleven months in the study. The control ponds and the antifungal-treated ponds are in the left and right columns, respectively. The 2010 and 2011 cohorts are shown in white and black, respectively. Fungicide treatment was applied to tadpoles in the months of May, June, July, and September (asterisks). Uninfected tadpoles were not included in the calculation of the mean *Bd* zoospore load. The bars show the 95% confidence limits of the mean.

The analysis of the *Bd* zoospore load data was restricted to the subset of infected tadpoles that had a *Bd* zoospore load > 0.01 (n = 1638 tadpoles). The seasonal pattern in the *Bd* zoospore load resembled the seasonal pattern of the *Bd* prevalence (Fig. 2). For the 2010 cohort, the *Bd* zoospore load remained stable or declined from May to July. For the 2011 cohort, the *Bd* zoospore load increased from August to January, before declining from January to April (Fig. 2). Similar to Fig. 1, the mean *Bd* zoospore load in the three treatment ponds is based on all the infected tadpoles that were captured, including those that had never been treated with fungicide.

### Effect of the fungicide treatment on the *Bd* zoospore load in tadpoles at the pond level

We used linear mixed effects models (LME) with normal errors to model the log10-transformed *Bd* zoospore load as a function of the fungicide treatment (control, treated), the covariate time, and their interaction. All tadpoles belonging to the same pond were assigned the same fungicide treatment status (i.e. statistical analysis was done at the pond level). The 2010 and 2011 cohorts were analysed separately because they occurred at different times of the year. The interpretation of the parameter estimates is the same as described for the prevalence of *Bd*. The intercept date for the 2010 cohort is May 5, 2011, whereas the intercept date for the 2011 cohort is December 27, 2011 (see statistical methods for details and Table S1).

### Effect of the fungicide treatment on the *Bd* zoospore load in the 2010 cohort of tadpoles at the pond level

For the subset of *Bd*-infected tadpoles in the 2010 cohort, examination of the model selection results (Tables 3 and S6) and the parameter estimates (Table S7) found no evidence that the dynamics of the *Bd* zoospore load differed between pond types or that the *Bd* zoospore load changed over time. Similarly, there was no evidence of any pre-existing differences in the *Bd* zoospore load between the two types of ponds prior to any application of the fungicide treatment to the 2010 cohort (Table S7).

**Table 3:** Model selection results are shown for the log10-transformed *Bd* zoospore load of the 2010 cohort of *A. obstetricans* tadpoles.

ID	F	T	F:T	Random	Df	logLik	AIC	Δ AIC	Weight 1	Weight 2
20	+	+	+	p + m + p:m	8	−1056.5	2129.4	0	0.86	0.86
16	+	+		p + m + p:m	7	−1060	2134.2	4.8	0.08	0.94
12		+		p + m + p:m	6	−1061.3	2134.8	5.4	0.06	1.00

The log10-transformed *Bd* zoospore load was modelled as a linear mixed effects model with normal errors. Of the 20 models in the set, only the top 3 models are shown for which the cumulative support (Weight 2) ≥95.0%. Shown for each model are: the model ID (ID), the fixed effects structure (F = fungicide treatment, T = t﻿ime, and F:T = interaction), the random effects structure (Random; p = pond identity, m = month identity, p:m = interaction), the model degrees of freedom (Df), the log-likelihood (logLik), the Akaike information criterion (AIC), the difference in the AIC value from the top model (Δ AIC), the model weight (Weight 1), and the cumulative weight (Weight 2). Table S6 shows the results from the full model selection.

### Effect of the fungicide treatment on the *Bd* zoospore load in the 2011 cohort of tadpoles at the pond level

For the subset of *Bd*-infected tadpoles in the 2011 cohort, examination of the parameter estimates found no evidence that the dynamics of the *Bd* zoospore load differed between pond types (Tables 4 and S8). There was strong support for a positive quadratic effect of time (support = 99.9% in Tables 4 and S8; slope = 27.87; 95% CI = 16.07 to 39.67; Table S9), indicating that *Bd* zoospore load was higher in the winter compared to the previous summer or the subsequent spring in both pond types (Fig. 2). Finally, the contrast of the Y-intercept suggests that on December 27, 2011, the *Bd* zoospore load was higher in the treated ponds than the control ponds (contrast of Y-intercept = 0.92; 95% CI = 0.40 to 1.45; Table S9).

**Table 4:** Model selection results are shown for the log10-transformed *Bd* zoospore load of the 2011 cohort of *A. obstetricans* tadpoles.

ID	F	T	T^2^	F:T	F:T^2^	Random	Df	logLik	AIC	Δ AIC	Weight 1	Weight 2
36	+	+	+	+	+	p + m + p:m	10	−2375.9	4772.1	0.00	0.855	0.86
32	+	+	+	+		p + m + p:m	9	−2378.8	4775.8	3.77	0.130	0.99

The log10-transformed *Bd* zoospore load was modelled as a linear mixed effects model with normal errors. Of the 36 models in the set, only the top 2 models are shown for which the cumulative support (Weight 2) ≥95.0%. Shown for each model are: the model ID (ID), the fixed effects structure (F = fungicide treatment, T = time, T^2^ = quadratic effect of time, F:T = inter﻿action, and F:T^2^ = interaction), the random effects structure (Random; p = pond identity, m = month identity, p:m = interaction), the model degrees of freedom (Df), the log-likelihood (logLik), the Akaike information criterion (AIC), the difference in the AIC value from the top model (Δ AIC), the model weight (Weight 1), and the cumulative weight (Weight 2). Table S8 shows the results from the full model selection.

### Effect of the fungicide treatment on the *Bd* zoospore load at the individual level

In the three treatment ponds (SagO, SagU, Herg), tadpoles were marked with visible implant elastomer (VIE) tags to indicate whether they had been treated with fungicide once or twice. The VIE tags allowed us to test whether the number of times that an individual tadpole had been treated with fungicide influenced its *Bd* zoospore load. For this reason, these analyses were considered as occurring at the individual level rather than the pond level. The analyses were done separately for the following three capture sessions: June 2011 (recapture of 2010 cohort tadpoles marked 0 or 1 times in May 2011), July 2011 (recapture of 2010 cohort tadpoles marked 0, 1, or 2 times in May and/or June 2011), and October 2011 to April 2012 (recapture of 2011 cohort tadpoles marked 0 or 1 times in September 2011).

**Figure 3:**
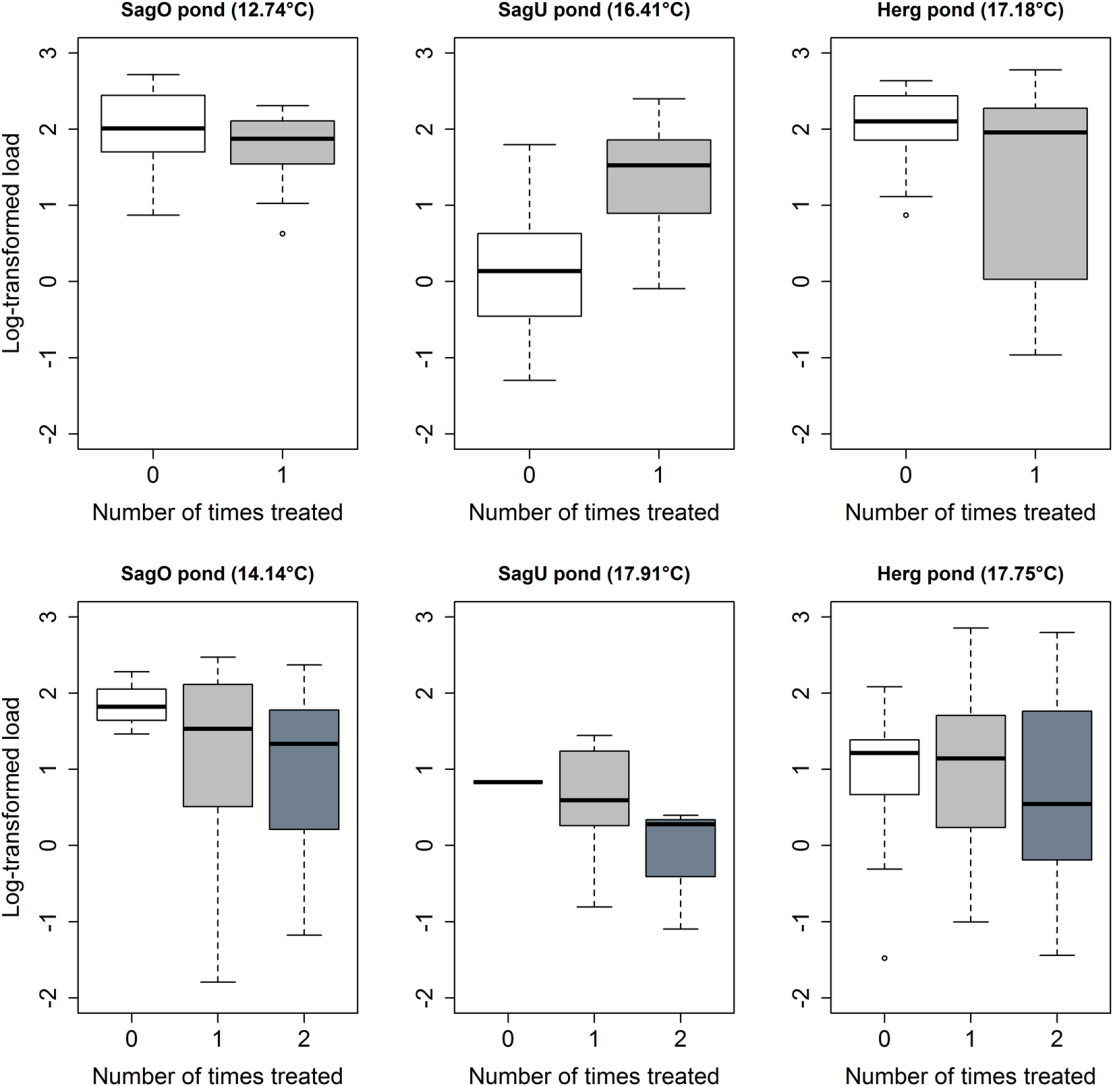
For the 2010 cohort of *A. obstetricans* tadpoles captured in June 2011 (top row) and July 2011 (bottom row), the log10-transformed *Bd* zoospore loads are shown as a function of the number of fungicide applications to individual tadpoles for each of the three treated ponds. The mean pond temperatures for the 30 days prior to capture are shown in brackets after the pond name. Shown are the medians (black lines), the upper and lower quartiles (edges of the box), the minimum and maximum values (whiskers), and the outliers (circles) for each pond.

For the 2010 cohort tadpoles captured in June 2011, the interaction between pond and fungicide treatment was significant (F_2, 113_ = 18.29, p < 0.001). The fungicide treatment decreased the *Bd* zoospore load of the tadpoles in the SagO and Herg ponds but increased the *Bd* zoospore load in the SagU pond (top row of Fig. 3). For the 2010 cohort tadpoles captured in July 2011, the fungicide treatment reduced the *Bd* zoospore load in individual tadpoles (bottom row of Fig. 3) but the effect was not significant (F_1,121_ = 2.155, p = 0.145). Similarly, for the 2011 cohort tadpoles, the fungicide reduced the *Bd* zoospore load in individual tadpoles but the effect was not significant (F_1,559_ = 1.819, p = 0.178). We point out that in Fig. 3, the ranking of the median *Bd* zoospore load follows the expected pattern in 5 of the 6 panels (the SagU pond in the top row is the exception). In summary, tadpoles that had been treated with fungicide once or twice, generally had lower zoospore loads than the tadpoles that had never been treated but the effect was not significant.

### Effects of temperature and time on the *Bd* infection in the 2011 cohort of tadpoles

We had originally wanted to include pond temperature as a covariate in the preceding analyses. Unfortunately, the temperature data logger failed for the SchU pond. In the preceding analysis, we decided that it was more important to include the SchU pond than temperature. The purpose of the present analysis was to estimate the effects of temperature on *Bd* infection. This analysis was restricted to the 2011 cohort and we did not re-examine the effect of the fungicide treatment. There is a strong relationship between temperature and day of the year (time) and it is therefore a challenge to separate their effects. We tried to model the temporal dynamics of *Bd* infection as thoroughly as possible to make the analysis as conservative as possible with respect to detecting an effect of temperature (see the statistical methods).

### Effects of temperature and time on the *Bd* prevalence in the 2011 cohort of tadpoles

For the *Bd* prevalence of the 2011 cohort, there was strong support for a positive linear effect of time (support = 85.0% in Table S10; mean = 48.7; 95% CI = 1.1 to 96.3; Table S11), indicating that *Bd* prevalence increased over time. In contrast, there was not much support that the quadratic effect of time and the linear and quadratic effects of temperature influenced the *Bd* prevalence of the 2011 cohort (Tables S10 and S11).

### Effects of temperature and time on the *Bd* zoospore load in the 2011 cohort of tadpoles

For the *Bd* zoospore load of the 2011 cohort, the top model had 98.7% of the support and included the linear and quadratic effects of time and the linear effect of temperature (Table S12). The positive quadratic effect of time indicated that the *Bd* zoospore load was higher in the winter compared to the previous summer or the subsequent spring (Table S13). The positive effect of temperature (mean = 23.8; 95% CI = 2.0 to 45.6; Table S13) indicated that after controlling for time, higher temperatures increased the *Bd* zoospore load in tadpoles of the 2011 cohort.

## Discussion

Developing treatments against fungal diseases is a priority for both humans and wildlife^9–12, 32^. While it is in principle possible to eradicate wildlife diseases in nature^14, 33^, a more realistic goal is to reduce the negative effects of the pathogen on host populations^12^. Hudson *et al*.^15^ showed that antifungal treatments can reduce *Bd* infection of wild frogs and increase their survival. Our field experiment shows that it is possible to transiently reduce *Bd* prevalence in tadpole populations using antifungal agents and a capture-treat-release approach. As shown by the significant treatment-by-time interaction, the antifungal treatment reduced the prevalence of *Bd* in the 2010 cohort over the months of May, June, and July, whereas no such decline was observed in the control ponds (Fig. 1). Thus, the goal of reducing *Bd* prevalence in the 2010 cohort was achieved in the short term. Another interesting result was that the fungicide treatment of the 2010 cohort changed the subsequent dynamics of the *Bd* prevalence in the 2011 cohort. For the 2011 cohort in the control ponds, the *Bd* prevalence was high and did not change over time (from August 2011 to April 2012; Fig. 1). In contrast, for the 2011 cohort in the treated ponds, the *Bd* prevalence was low in August and September and had to increase rapidly over the fall to reach 100.0% prevalence in the winter (Fig. 1). This result is consistent with the interpretation that the antifungal treatment of the 2010 cohort (in May, June, and July) reduced transmission of *Bd* from this cohort to the 2011 tadpole cohort. Our study is in agreement with a previous mesocosm study showing that an antifungal treatment blocked *Bd* transmission from infected to uninfected *A. obstetricans* tadpoles^34^. Thus, future control strategies could attempt to block transmission (e.g., use of a fungicide to kill free-living zoospores) rather than clear infection.

The effect of the fungicide treatment on the *Bd* zoospore load was weaker and inconsistent (Fig. 2), which is similar to the results of a previous laboratory experiment^31^. While the fungicide treatment of the 2010 cohort reduced the *Bd* prevalence, there was no effect on the *Bd* zoospore load. While the fungicide treatment of the 2010 cohort changed the dynamics of the *Bd* prevalence in the 2011 cohort, there was no such effect on the *Bd* zoospore load. Counterintuitively, for the 2011 cohort on the intercept date (December 27, 2011), the *Bd* zoospore load was actually higher in the treated ponds than the control ponds. Overall, the results are consistent with the interpretation that the antifungal treatment cleared weak infections (low *Bd* zoospore load) but had no measurable effect on the heavy infections (high *Bd* zoospore load). The analysis at the individual level generally found that the median *Bd* zoospore load declined with the number of fungicide applications, but the results were not statistically significant (Fig. 3). One limitation with using PCR to estimate pathogen load is that this method cannot distinguish between dead and living cells. If the fungicide treatment kills a fraction of the *Bd* zoospore load, but the tadpole does not clear the dead *Bd* zoospores from its mouthparts in between capture sessions, then the *Bd* zoospore load will remain unchanged. Ideally, our estimates of pathogen load should count live and not dead cells.

Even though the antifungal treatment reduced *Bd* prevalence in the 2010 cohort and initially in the 2011 cohort, the treatment was unable to prevent the *Bd* infection from spreading through the 2011 cohort tadpoles over the course of the autumn and winter (i.e. after antifungal treatment had ended). By December, almost 100% of all the tadpoles were infected with *Bd* in both the control ponds and the treated ponds. The monotonic rise in the *Bd* prevalence from summer to winter suggests that there is a very high force of infection in these tadpole populations. The observation that *Bd* prevalence and *Bd* zoospore load remained high throughout the winter (Figs 1 and 2) suggests that the immune system of overwintering tadpoles^35^ is down regulated and cannot clear the *Bd* infection^36^.

Previous studies have shown that the epidemiology of *Bd* depends on temperature^37–41^. In the present study, temperature did not affect *Bd* prevalence but had a positive effect on *Bd* zoospore load. In the laboratory, the optimal temperature conditions for the growth of *Bd* are 17–23 °C^42^. In the present study, the mean pond temperatures ranged from 2.2–18.2 °C, and were generally below the optimal temperature conditions for *Bd* growth. When environmental temperatures are below the physiological optimum, we expect to find the observed positive relationship between temperature and *Bd* zoospore load. In contrast, if environmental temperatures are above the physiological optimum, we expect to find a negative relationship between temperature and *Bd* zoospore load. Our study shows that it is possible to disentangle the effect of temperature from the effect of season (modelled here as time and the quadratic effect of time). In the present study, the *Bd* infection spread rapidly during the months of August, September, and October, when pond temperatures were in the range of the optimal growth conditions (Figs 1 and 2; a similar temporal pattern was reported from Spain)^43^. During the late fall (November) and winter (December and January), the *Bd* zoospore load remained high but did not change in the tadpole population (Figs 1 and 2). Thus, one explanation is that cold winter temperatures simply preserved the *Bd* infection in the tadpoles in the same way that a freezer preserves *Bd*-infected tissue samples. More recent studies have shown that *Bd* maintains growth and zoospore production over a much wider temperature range than the laboratory optimum of 17–23 °C^44^. Thus an alternative explanation is that the *Bd* pathogen was actively growing and reproducing during the cold winter months but that this growth did not alter the zoospore load. The *Bd* zoospore load of the 2011 cohort appears to decrease from January to April (Fig. 2) suggesting that the tadpole immune system has become more active. In summary, the *Bd* infection rapidly invaded the 2011 cohort during the warmer temperatures of the summer and fall and reached 100% prevalence by winter. During the cold winter months, the *Bd* infection was preserved in the overwintering tadpoles and the *Bd* zoospore load did not change.

The present study has shown that a capture-treat-release approach using antifungals can be used to transiently decrease the prevalence of *Bd* in natural tadpole populations. The treatment effect might have been greater if we had used a more effective antifungal agent such as Itraconazole^15^ but we opted for an antifungal agent that is easily available and has a simple application^31^. For the control strategy of any wildlife disease, it is generally unlikely that infection can be cleared from all individuals in nature. The successful control of any pathogen depends on the proportion of host individuals that has to be treated such that the pathogen goes extinct. The proportion of individuals which has to be treated depends on the basic reproduction rate, R_0_, of the pathogen and is given by p > 1 − (1/R_0_)^33^. Lam *et al*.^45^ assumed that p ≈ 0.8 which led Woodhams *et al*.^9^ to suggest that R_0_ ≈ 5 for *Bd*. Since R_0_ for *Bd* is unknown, it is hard to predict whether successful control of *Bd* might be achieved (reservoir hosts and persistence of zoospores in the environment make control even more difficult). Thus, estimating R_0_, a basic epidemiological parameter, would be of great importance to better inform the science and practice of disease control.

Many potential anti-*Bd* treatments have been tested in the laboratory^9–12^ but the number of successful attempts at *Bd* mitigation is still very low^12^. It is therefore important to do more field trials of mitigation methods^14, 15^ that are ethically and legally acceptable^9, 12^. Since the *Bd*-host interaction is strongly context-dependent^46^, mitigation strategies should be tested under a variety of environmental conditions to determine whether a method can be used only locally or whether it is transferrable to other systems. Last but not least, even if recent studies suggest that *Bd* mitigation in natural populations may be possible, prevention is better than cure^47^. Thus, preventing the emergence of pathogens in naïve wildlife populations is the best way to protect biodiversity against emerging infectious diseases^48^.

## Materials and Methods

### Study location

The study was carried out in canton Lucerne in central Switzerland. At four different sites, six ponds that were known to contain *A. obstetricans* and *Bd* ^30^ were selected: Sagerhüsli upper (SagO), Sagerhüsli lower (SagU), Hergiswald (Herg), Schauensee upper (SchO), Schauensee lower (SchU), and Hinter Rohren (HiR). These ponds were similar in elevation (range = 558–798 m), surface area (6–30 m^2^), and depth (0.5–2.5 m). The water temperature of each of the six ponds was measured six times per day over the duration of the study using HOBO H8 temperature data loggers (Onset Computer Corporation, Bourne MA, USA). The tadpoles in SagO, SagU, and Herg were treated with an antifungal agent whereas SchO, SchU, and HiR served as control ponds.

### Experimental design

The study took place from May 2011 to April 2012 and involved two different cohorts of tadpoles: 2010 and 2011. For the 2010 cohort, most of the tadpoles (98.2% = 612/623) were captured in the months of May, June, and July (first capture date = May 5, 2011, last capture date = July 11, 2011) and this cohort left the ponds as metamorphs by August 2011. For the 2011 cohort, most of the tadpoles (98.5% = 1484/1507) were captured from August 2011 to April 2012 (first capture date = August 2, 2011, last capture date = April 3, 2012). Tadpoles in the 2010 cohort were treated with fungicide on three separate occasions (May, June, and July), whereas tadpoles in the 2011 cohort were treated once (September). Field-captured tadpoles were treated with General Tonic^®^ antifungal agent for 8 days in 100 L cattle tanks that were filled with 50 L of tap water. We used a concentration of 0.6 ml of General Tonic^®^ per litre of water^31^. After 4 days, the tanks were filled with a fresh solution of General Tonic^®^ water. Cattle tanks were placed near the ponds and covered with a shade cloth. Fungicide-treated tadpoles were marked with visible implant elastomer (VIE) tags before being released into their original ponds^49^. A different VIE tag colour was used for the fungal treatments of May, June, July and September 2011. Tadpoles were tagged every time that they were captured and treated with antifungals.

### Survey of *Bd* infection in *Alytes obstetricans* tadpoles

Over the duration of the study (May 2011 to April 2012), *A. obstetricans* tadpoles were captured each month except in February 2012 because the ponds were frozen. Tadpoles were captured using dip nets and tested for *Bd* infection by swabbing the mouthparts with sterile rayon-tipped plain swabs using a plastic applicator (Copan, Brescia, Italy). In the treatment ponds, the surveyors swabbed a maximum of 30 previously treated tadpoles (VIE tag present) and 30 untreated tadpoles (no VIE tag) each month. In the control ponds, the surveyors swabbed a maximum of 30 untreated tadpoles each month. We obtained a total of 2132 mouthpart swab samples that were stored at −20 °C until analysis.

### DNA extractions of the tadpole mouthpart swabs

DNA was extracted from the tadpole mouthpart swabs using the PrepMan^®^ Ultra Sample Preparation Reagent Protocol (Applied Biosystems by Life Technologies). The tip of each *Bd* swab (1–2 mm) was cut off using a sterile and single-use surgical carbon blade (Paramount^®^). The tips were placed into 60 µL of PrepMan^TM^ Ultra (Applied Biosystems by Life Technologies) with 0.03–0.04 g Zirconial/Glas-beads (0.5 mm diameter, Carl Roth). The samples were shaken at 30 Hz for 45 seconds, using a TissueLyser Adapter Set 2 × 24 (QIAGEN^®^) then centrifuged at 13,000 rpm for 1 minute using an ALC Multispeed refrigerated centrifuge. The last two steps were repeated before placing the samples into an Eppendorf Thermomixer Comfort^®^ at 99 °C for 10 minutes followed by centrifugation at 13,000 rpm for 3 minutes^47^. The resultant supernatant was placed into an Eppendorf tube and stored at −20 °C.

### Quantitative PCR to determine the *Bd* zoospore load

Quantitative PCR (qPCR) was used to estimate the zoospore load of *Bd* in the tadpole mouthpart swabs following the protocol of Boyle *et al*.^50^. The specific primers ITS1–3 Chytr and 5.8S Chytr and the minor groove binder probe Chytr MGB2 were used to amplify and detect the 5.8S rRNA gene and the flanking internal transcribed spacer (ITS) of the *Bd* pathogen^50^. Each qPCR reaction had a total reaction volume of 20 µL and contained 10 µL of 2x Master Mix (FastStart Essential DNA Probes Master, Roche Applied Science), 0.9 µM of each primer, 0.25 µM of the MGB2 probe, and 5 µL of DNA (diluted 1:10 in water). The thermocycling conditions were as follows: a preincubation step at 95 °C for 10 minutes followed by 50 cycles at 95 °C for 15 seconds and 60 °C for 1 minute^50^. The qPCR was performed using a LightCycler^®^ 96 Real-Time PCR System (Roche Applied Science). All DNA samples were run in duplicate. Each 96-well plate contained 3 negative controls, 3 positive controls, and 4 standards (see supplemental material) run in triplicate to quantify the *Bd* zoospore load. The four standards contained 10^1^, 10^2^, 10^3^, and 10^4^ copy numbers of the target gene.

### Repeatability of the estimate of the *Bd* zoospore load

The repeatability of the *Bd* zoospore load was very high for a sample of 606 DNA extractions that had been run in duplicate for the qPCR assay (repeatability = 0.999, F_605, 606_ = 103.500, p < 0.001).

## Statistical Methods

### Effect of the fungicide treatment on the *Bd* prevalence and *Bd* zoospore load at the pond level

The 36 tadpoles that were captured outside the dominant sampling period for their cohort (13 tadpoles for 2010 cohort and 23 tadpoles for the 2011 cohort) were eliminated from the analysis. The final sample size was therefore 2096 tadpoles: 612 and 1484 tadpoles for the 2010 and 2011 cohorts, respectively. Tadpoles with a *Bd* zoospore load > 0.01 were defined as being infected with *Bd*. We analysed two different response variables: the proportion of tadpoles infected with *Bd* (n = 2096 tadpoles), and the log10-transformed *Bd* zoospore load for the subset of infected tadpoles (n = 1638 tadpoles). We used generalized linear mixed effects (GLME) models with binomial errors and linear mixed effects models (LME) with normal errors to model the *Bd* prevalence and the log10-transformed *Bd* zoospore load, respectively. The 2010 and 2011 cohorts were analysed separately because they occurred at different times of the year (i.e. we cannot use the same time scale for the two cohorts). The fixed effects structure included the fungicide treatment (control, treated), the covariate time, the covariate time^2^ (only for the 2011 cohort), and their interactions. For the 2011 cohort, the covariate time^2^ was included because the temporal change in the *Bd* infection resembled a negative quadratic function. The random effects structure included the categorical factors pond, month, and their interaction. In this study, we expected that there would be no difference between ponds at the beginning of the experiment. The fungicide treatment was expected to gradually reduce the *Bd* prevalence over time in the tadpoles. Thus, the treatment-by-time interaction was expected to be more informative than the main effect of treatment.

### Scaling of the time covariate

Each cohort had its own time scale: day 1 (May 5, 2011) to day 68 (July 11, 2011) for the 2010 cohort, and day 1 (August 2, 2011) to day 246 (April 3, 2012) for the 2011 cohort. For each cohort, the covariate time was expressed in months by dividing the number of days by 30. For the 2011 cohort, time and time^2^ were highly correlated, which caused problems with model convergence and parameter estimation. These variables were therefore transformed using the poly() function in R so that they were orthogonal to each other. The origin (t = 0) of this transformed time scale is day 136 (December 15, 2011; Table S1) for the *Bd* prevalence data (i.e., all the tadpoles in the 2011 cohort), and day 148 (December 27, 2011; Table S1) for the *Bd* zoospore load data (i.e., the subset of *Bd*-infected tadpoles). After transformation, time^2^ was a negative quadratic (concave down). A positive slope for the transformed time^2^ variable therefore meant that the *Bd* prevalence (or *Bd* zoospore load) was highest in the winter and lower in the fall and spring.

### Predictions

We monitored the epidemiology of the *Bd* infection (*Bd* prevalence and *Bd* zoospore load) over time. We expected the dynamics of the *Bd* infection to be different between the treatment ponds and the control ponds. In other words, we expected an interaction between the fungicide treatment and time, which indicates that the rate at which tadpoles acquire or lose *Bd* infections is different between the treatment ponds and the control ponds. In this study, we expected that the interaction term would be more important than the main effect of the fungicide treatment. For each cohort, the meaning of the contrast in the Y-intercept between the control and treated ponds depends on how the time covariate was rescaled. For the 2010 cohort, this contrast measures pre-existing differences in *Bd* infection between the two types of ponds on May 5, 2011, which was prior to the application of the fungicide treatment. For the 2011 cohort, this contrast compares the *Bd* prevalence (or log10-transformed *Bd* zoospore load) between treated and control ponds on December 15, 2011 (or on December 27, 2011).

### Model selection approach

We used a model selection approach based on the Akaike information criterion (AIC) to find the most parsimonious model. Models were ranked according to their AIC values and the Akaike weights were calculated for each model. The support for a given explanatory variable of interest is the sum of the Akaike weights of all the models in the set that include that particular explanatory variable. The support for a given explanatory variable can range from low (0.0%) to high (100.0%). We also used the Akaike weights to calculate the model-averaged parameter estimates and their 95% confidence limits^51^.

### Effect of the fungicide treatment on the *Bd* zoospore load at the individual level

In the three treatment ponds (SagO, SagU, Herg), the marking of the tadpoles with VIE tags allowed us to test whether the number of times that an individual tadpole had been treated with fungicide influenced its *Bd* zoospore load. We used linear models to test whether the number of fungicide treatments applied to an individual tadpole influenced its log10-transformed *Bd* zoospore load. This analysis was restricted to the tadpoles in the three experimental ponds (SagO, SagU, Herg). The 2010 and 2011 cohorts were analysed separately. For the 2010 cohort, the analysis was further split for the months of June and July because the number of fungicide applications differed between these two months. For the 2010 cohort, tadpoles captured in June (n = 172) had been treated never or once, whereas the tadpoles captured in July (n = 167) had been treated never, once, or twice. For the 2011 cohort, tadpoles captured in the months following the single fungicide treatment in September (n = 569) had been treated never or once.

### Effect of temperature on *Bd* infection in the 2011 cohort of tadpoles

We used GLME and LME models with binomial and normal errors to model *Bd* prevalence and the log10-transformed *Bd* zoospore load as a quadratic function of the pond temperature (°C) and a quadratic function of time (days). The random effects structure was similar to the previous models. We restricted these analyses to the 2011 cohort because there were only 3 time points for the 2010 cohort. Due to a data logger failure, the analysis was restricted to five ponds because mean monthly pond temperature data were not available for SchU. As before, time (range: day 1 to day 246) and time^2^ were rescaled as orthogonal contrasts, and the same was done for temperature (range: 2.2 °C to 18.2 °C) and temperature^2^. For the 2011 cohort, the origins of the transformed time scale and t﻿he transformed temperature scale are day 137 (December 16, 2011) and 9.48 °C (Table S1) for all the tadpoles (i.e., for the analysis of the *Bd* prevalence data), and day 151 (December 29, 2016) and 8.75 °C (Table S1) for the subset of *Bd*-infected tadpoles (i.e., for the analysis of the *Bd* zoospore load data). After transformation, time^2^ was a negative quadratic (Figure S1) whereas temperature^2^ was a positive quadratic (Figure S2). A positive slope for the transformed time^2^ variable therefore meant that the *Bd* prevalence (or *Bd* zoospore load) was highest in the winter and lower in the fall and spring (the reverse was true for the transformed temperature^2^ variable).

### Software package used for statistical analyses

We used R for all statistical analyses^52^. We used the glmer() function and the lmer() function in the lme4 package to run the generalized linear mixed effects models with binomial errors and normal errors, respectively. We used the model.sel() function and the model.av() function in the MuMIn package to create the model selection tables and the model-averaged parameter estimates. We used the confint() function in the base package to calculate the 95% confidence intervals (CI) for the model-averaged parameter estimates. We used the poly() function in the base package to rescale time and time^2^ (or temperature and temperature^2^) so that they were uncorrelated with each other.

### Ethics statement and animal experimentation permits

Experiments were performed in accordance with the relevant regulations (Tierschutzgesetz; Bundesgesetz über den Natur- und Heimatschutz). The study was conducted under the animal experimentation permit number 75/2009 by the veterinary office of the canton Zürich; the permit included an approval of the methods. Capture permits (as required by the nature conservation law) were provided by the nature conservation office of canton Lucerne.

## Electronic supplementary material

Supplemental Material
