# Supplementary material for: Antifungal treatment of wild amphibian populations caused a transient reduction in the prevalence of the fungal pathogen, Batrachochytrium dendrobatidis
Source: Sci Rep. 2017 Jul 20;7:5956. doi: 10.1038/s41598-017-05798-9 (PMC5519715; doi:10.1038/s41598-017-05798-9)
Supplement: Supplementary file 1 — Supplemental Material [file 41598_2017_5798_MOESM1_ESM.doc]

Title: Antifungal treatment of wild amphibian populations caused a transient reduction in the prevalence of the fungal pathogen, *Batrachochytrium dendrobatidis*

Authors: Corina C. Geiger, Cindy Bregnard, Elodie Maluenda, Maarten J. Voordouw, Benedikt R. Schmidt

Supplemental Material

**Additional molecular methods**

**PCR and sequencing of the 5.8S rRNA gene of the *Bd* pathogen.** The specific primers ITS1-3 Chytr and 5.8S Chytr (Boyle *et al*, 2004) were used to amplify the 5.8S rRNA gene and the flanking internal transcribed spacer (ITS). Amplification reactions consisted of 1X of Green GoTaq® Reaction Buffer, 0.5 mM of dNTPs, 0.9 µM of each primer, 1 U/µL of GoTaq® polymerase, and 5 µL of DNA in a total reaction volume of 25 µL. The thermocycling conditions were as follows: an initial denaturation step at 94°C for 2 minutes followed by 40 cycles of 94°C for 30 seconds, 59°C for 45 seconds, and 72°C for 30 seconds, and a final extension step at 72°C for 10 minutes (Annis *et al*, 2004). PCR amplicons were purified using a Wizard® SV Gel and PCR Clean-Up System kit (Promega) and were sequenced by Microsynth AG. Sequence identity was confirmed by blasting the sequences in the National Center for Biotechnology Information (NCBI).

**Standards for quantifying the *Bd* zoospore load.** The 5.8S rRNA gene and the flanking ITS gene were amplified using the conventional PCR protocol described above. The amplicon was ligated into the pCR®-TOPO®TA vector (Invitrogen^TM^ by Life Technologies^TM^). Ligation reactions consisted of 1 µL of salt solution, 1 µL of DNase-free water, 1 µL of pCR®-TOPO®TA vector, and 3 µL of amplicon. Two µL of the ligation mixture was added into a vial of One Shot® Chemically Competent *E. coli*. The ligation mixture was incubated for 25 minutes on ice before being warmed up at 42°C for 30 seconds in a mixing block. The ligation mixture was immediately transferred to ice and 250 µL of SOC medium was added to the mixture. The mixture was horizontally shaken at 37°C for one hour. The transformed cells were cultured overnight at 37°C on agar plates containing LB media, the antibiotic ampicillin (50 µg/mL), and 40 µL of the marker X-Gal for distinguishing between colonies that contained the insert (white) or not (blue). Plasmid DNA was extracted and purified using a Wizard® Plus SV Minipreps DNA Purification System (Promega). The DNA concentration was measured with a Thermo Scientific NanoDrop 2000 spectrophotometer. The size of the recombinant plasmid (3956 nucleotides) was used to calculate the number of *Bd* gene copies in the plasmid mini-prep. The mini-prep was diluted to create a series of eight standards that contained 10^1^, 10^2^, 10^3^, 10^4^, 10^5^, 10^6^, 10^7^, and 10^8^ copies of the targeted *Bd* gene.

**Table S1. Time and temperature were rescaled for the 2011 cohort of *A. obstetricans* tadpoles to avoid co-linearity problems between the linear and quadratic terms.** (A) Rescaling of the time covariate for the analysis of the effect of the fungicide treatment. (B) Rescaling of the time and temperature covariates for the analysis of the effect of the temperature.

| **(A) Fungicide treatment** | **Prevalence** | **Log-transformed load** |
| --- | --- | --- |
| Time 0 after rescaling | Day 136  (December 15, 2011) | Day 148  (December 27, 2011) |
| **(B) Temperature** | **Prevalence** | **Log-transformed load** |
| Time 0 after rescaling | Day 137  (December 16, 2011) | Day 150  (December 29, 2011) |
| Temperature 0 after rescaling | 9.48°C | 8.75°C |

**Table S2. Model selection results are shown for the *Bd* prevalence of the 2010 cohort of *A. obstetricans* tadpoles.** The *Bd* prevalence was modelled as a generalized linear mixed effects model with binomial errors. Fixed factors included fungicide treatment (F), the covariate time (T), and their interaction (F:T). Random factors included pond (p), month (m), and their interaction (p:m). Shown for each model are: the model ID (ID), the fixed effects structure (F, T, and F:T), the random effects structure (Random), the model degrees of freedom (Df), the log-likelihood (logLik), the Akaike information criterion (AIC), the difference in the AIC value from the top model (Δ AIC), the model weight (weight 1), and the cumulative weight (weight 2).

| **ID** | **F** | **T** | **F:T** | **Random** | **Df** | **logLik** | **AIC** | **Δ AIC** | **Weight 1** | **Weight 2** |
| --- | --- | --- | --- | --- | --- | --- | --- | --- | --- | --- |
| 1 | + | + | + | p | 5 | -313.4 | 636.8 | 0.00 | 0.622 | 0.62 |
| 2 | + | + | + | p+m | 6 | -313.4 | 638.9 | 2.04 | 0.224 | 0.84 |
| 3 | + | + | + | p+m+p:m | 7 | -313.0 | 640.3 | 3.44 | 0.111 | 0.95 |
| 4 |  |  |  | p+m+p:m | 4 | -318.7 | 645.4 | 8.55 | 0.009 | 0.96 |
| 5 |  | + |  | p+m+p:m | 5 | -317.7 | 645.5 | 8.66 | 0.008 | 0.97 |
| 6 |  | + |  | p | 3 | -320.1 | 646.1 | 9.30 | 0.006 | 0.98 |
| 7 | + | + | + | m | 5 | -318.3 | 646.8 | 9.95 | 0.004 | 1.00 |
| 8 | + |  |  | p+m+p:m | 5 | -318.5 | 647.0 | 10.20 | 0.004 | 1.00 |
| 9 | + | + |  | p+m+p:m | 6 | -317.5 | 647.2 | 10.38 | 0.003 | 1.00 |
| 10 | + | + |  | p | 4 | -319.9 | 647.9 | 11.06 | 0.002 | 1.00 |
| 11 |  | + |  | p+m | 4 | -320.1 | 648.2 | 11.33 | 0.002 | 1.00 |
| 12 |  |  |  | p | 2 | -322.7 | 649.4 | 12.57 | 0.001 | 1.00 |
| 13 | + | + |  | p+m | 5 | -319.9 | 649.9 | 13.09 | 0.001 | 1.00 |
| 14 |  |  |  | p+m | 3 | -322.2 | 650.4 | 13.58 | 0.001 | 1.00 |
| 15 | + |  |  | p | 3 | -322.6 | 651.2 | 14.33 | 0.000 | 1.00 |
| 16 | + |  |  | p+m | 4 | -322.0 | 652.1 | 15.26 | 0.000 | 1.00 |
| 17 |  | + |  | m | 3 | -324.8 | 655.6 | 18.78 | 0.000 | 1.00 |
| 18 | + | + |  | m | 4 | -324.2 | 656.6 | 19.72 | 0.000 | 1.00 |
| 19 |  |  |  | m | 2 | -326.7 | 657.3 | 20.49 | 0.000 | 1.00 |
| 20 | + |  |  | m | 3 | -326.2 | 658.4 | 21.52 | 0.000 | 1.00 |

**Table S3. Model-averaged parameter estimates are shown for the *Bd* prevalence of the 2010 cohort of *A. obstetricans* tadpoles.** Shown are the parameter types, the parameter names, the parameter estimates, and the 95% confidence limits (LL = lower limit and UL = upper limit). Estimate 1 is averaged over all the models in the set. Estimate 2 is averaged over the subset of models with a cumulative support of 95%. The 95% confidence limits are for estimate 2.

| **Type** | **Name** | **Estimate 1** | **Estimate 2** | **95% LL** | **95% UL** |
| --- | --- | --- | --- | --- | --- |
| Intercept^a^ | Control ponds | 1.148921 | 1.148921 | 0.559152 | 1.738690 |
| Contrast1^b^ | Fungicide ponds | 0.225341 | 0.231562 | -0.571439 | 1.034563 |
| Slope^c^ | Time | 4.895243 | 4.970345 | -3.757657 | 13.698348 |
| Contrast2^d^ | Fungicide:Time | -18.76909 | -19.51690 | -30.664814 | -8.368987 |

^a^ Logit value of the intercept of the control ponds on May 5, 2011

^b^ Logit value of the contrast1[intercept(fungicide) – intercept(control)] on May 5, 2011

^c^ Logit value of the slope of *Bd* prevalence versus time in the control ponds

^b^ Logit value of the contrast2[slope(fungicide) – slope(control)]

**Table S4. Model selection results are shown for the *Bd* prevalence of the 2011 cohort of *A. obstetricans* tadpoles.** The *Bd* prevalence was modelled as a generalized linear mixed effects model with binomial errors. Fixed factors included fungicide treatment (F), the linear term of time (T), the quadratic term of time (T^2^), and their interactions (F:T; F:T^2^). Random factors included pond (p), month (m), and their interaction (p:m). Shown for each model are: the model ID (ID), the fixed effects structure (F, T, T^2^, F:T, and F:T^2^), the random effects structure (Random), the model degrees of freedom (Df), the log-likelihood (logLik), the Akaike information criterion (AIC), the difference in the AIC value from the top model (Δ AIC), the model weight (weight 1), and the cumulative weight (weight 2).

| **ID** | **F** | **T** | **T^2^** | **F:T** | **F:T^2^** | **Random** | **Df** | **logLik** | **AIC** | **Δ AIC** | **Weight 1** | **Weight 2** |
| --- | --- | --- | --- | --- | --- | --- | --- | --- | --- | --- | --- | --- |
| 32 | + | + | + | + |  | p+m+p:m | 8 | -575.9 | 1167.9 | 0.00 | 0.237 | 0.24 |
| 16 |  | + | + |  |  | p+m+p:m | 6 | -578.0 | 1168.1 | 0.16 | 0.219 | 0.46 |
| 36 | + | + | + | + | + | p+m+p:m | 9 | -575.5 | 1169.1 | 1.16 | 0.132 | 0.59 |
| 24 | + | + |  | + |  | p+m+p:m | 7 | -577.6 | 1169.3 | 1.36 | 0.120 | 0.71 |
| 28 | + | + | + |  |  | p+m+p:m | 7 | -577.8 | 1169.7 | 1.74 | 0.099 | 0.81 |
| 12 |  | + |  |  |  | p+m+p:m | 5 | -579.9 | 1169.8 | 1.90 | 0.092 | 0.90 |
| 20 | + | + |  |  |  | p+m+p:m | 6 | -579.7 | 1171.4 | 3.47 | 0.042 | 0.94 |
| 04 |  |  |  |  |  | p+m+p:m | 4 | -581.7 | 1171.4 | 3.53 | 0.041 | 0.98 |
| 08 | + |  |  |  |  | p+m+p:m | 5 | -581.5 | 1173.0 | 5.09 | 0.019 | 1.00 |
| 31 | + | + | + | + |  | l+m | 7 | -600.5 | 1215.1 | 47.21 | 0.00 | 1.00 |
| 23 | + | + |  | + |  | l+m | 6 | -602.1 | 1216.2 | 48.25 | 0.00 | 1.00 |
| 35 | + | + | + | + | + | l+m | 8 | -600.2 | 1216.5 | 48.59 | 0.00 | 1.00 |
| 15 |  | + | + |  |  | l+m | 5 | -607.0 | 1224.0 | 56.11 | 0.00 | 1.00 |
| 27 | + | + | + |  |  | l+m | 6 | -606.4 | 1224.9 | 57.00 | 0.00 | 1.00 |
| 11 |  | + |  |  |  | l+m | 4 | -609.0 | 1226.0 | 58.10 | 0.00 | 1.00 |
| 19 | + | + |  |  |  | l+m | 5 | -608.4 | 1226.8 | 58.93 | 0.00 | 1.00 |
| 03 |  |  |  |  |  | l+m | 3 | -611.7 | 1229.3 | 61.43 | 0.00 | 1.00 |
| 07 | + |  |  |  |  | l+m | 4 | -611.1 | 1230.1 | 62.23 | 0.00 | 1.00 |
| 29 | + | + | + | + |  | m | 6 | -633.9 | 1279.9 | 112.00 | 0.00 | 1.00 |
| 21 | + | + |  | + |  | m | 5 | -635.5 | 1281.1 | 113.21 | 0.00 | 1.00 |
| 33 | + | + | + | + | + | m | 7 | -633.6 | 1281.2 | 113.27 | 0.00 | 1.00 |
| 25 | + | + | + |  |  | m | 5 | -639.1 | 1288.3 | 120.41 | 0.00 | 1.00 |
| 30 | + | + | + | + |  | p | 6 | -638.7 | 1289.5 | 121.60 | 0.00 | 1.00 |
| 17 | + | + |  |  |  | m | 4 | -641.2 | 1290.4 | 122.46 | 0.00 | 1.00 |
| 34 | + | + | + | + | + | p | 7 | -638.3 | 1290.7 | 122.76 | 0.00 | 1.00 |
| 05 | + |  |  |  |  | m | 3 | -643.9 | 1293.7 | 125.82 | 0.00 | 1.00 |
| 14 |  | + | + |  |  | p | 4 | -643.4 | 1294.9 | 126.94 | 0.00 | 1.00 |
| 26 | + | + | + |  |  | p | 5 | -642.6 | 1295.3 | 127.41 | 0.00 | 1.00 |
| 13 |  | + | + |  |  | m | 4 | -646.5 | 1301.0 | 133.09 | 0.00 | 1.00 |
| 09 |  | + |  |  |  | m | 3 | -649.2 | 1304.5 | 136.57 | 0.00 | 1.00 |
| 01 |  |  |  |  |  | m | 2 | -652.2 | 1308.5 | 140.57 | 0.00 | 1.00 |
| 22 | + | + |  | + |  | l | 5 | -666.3 | 1342.6 | 174.70 | 0.00 | 1.00 |
| 10 |  | + |  |  |  | l | 3 | -670.3 | 1346.6 | 178.71 | 0.00 | 1.00 |
| 18 | + | + |  |  |  | l | 4 | -669.5 | 1346.9 | 179.03 | 0.00 | 1.00 |
| 02 |  |  |  |  |  | l | 2 | -734.3 | 1472.6 | 304.71 | 0.00 | 1.00 |
| 06 | + |  |  |  |  | l | 3 | -733.6 | 1473.3 | 305.36 | 0.00 | 1.00 |

**Table S5. Model-averaged parameter estimates are shown for the *Bd* prevalence of the 2011 cohort of *A*. *obstetricans* tadpoles.** Shown are the parameters types, the parameters names, the parameter estimates, and the 95% confidence limits (LL = lower limit and UL = upper limit). Estimate 1 is averaged over all the models in the set. Estimate 2 is averaged over the subset of models with a cumulative support of 95%. The 95% confidence limits are for estimate 2.

| **Type** | **Name** | **Estimate 1** | **Estimate 2** | **95% LL** | **95% UL** |
| --- | --- | --- | --- | --- | --- |
| Intercept^a^ | Control ponds | 2.525817 | 2.525817 | 1.183205 | 3.868428 |
| Contrast1^b^ | Fungicide ponds | -0.258707 | -0.398545 | -1.968279 | 1.171188 |
| Slope1^c^ | Time | 28.00299 | 29.76468 | -4.169655 | 63.699009 |
| Contrast2^d^ | Fungicide:Time | 12.52007 | 25.56976 | 2.763422 | 48.376107 |
| Slope2^e^ | Time^2^g^ | 21.68188 | 31.56266 | 2.627170 | 60.498150 |
| Contrast3^f^ | Fungicide:Time^2 | 1.61952 | 12.22778 | -11.98107 | 36.436622 |

^a^ Logit value of the intercept of the control ponds on December 15, 2011

^b^ Logit value of the contrast1[intercept(fungicide) – intercept(control)] on Dec 15, 2011

^c^ Logit value of the slope1 of *Bd* prevalence versus time in the control ponds

^d^ Logit value of the contrast2[slope1(fungicide) – slope1(control)]

^e^ Logit value of the slope2 of *Bd* prevalence versus time^2 in the control ponds

^f^ Logit value of the contrast3[slope2(fungicide) – slope2(control)]

^g^ The transformed time^2 variable is a negative quadratic function. A positive slope2 means that the *Bd* prevalence is highest in the winter and lower in the fall and spring

**Table S6. Model selection results are shown for the log10-transformed *Bd* zoospore load of the 2010 cohort of *A. obstetricans* tadpoles.** The log10-transformed *Bd* zoospore load was modelled as a linear mixed effects model with normal errors. Fixed factors included fungicide treatment (F), the covariate time (T), and their interaction (F:T). Random factors included pond (p), month (m), and their interaction (p:m). Shown for each model are: the model ID (ID), the fixed effects structure (F, T, and F:T), the random effects structure (Random), the model degrees of freedom (Df), the log-likelihood (logLik), the Akaike information criterion (AIC), the difference in the AIC value from the top model (Δ AIC), the model weight (weight 1), and the cumulative weight (weight 2).

| **ID** | **F** | **T** | **F:T** | **Random** | **Df** | **logLik** | **AIC** | **Δ AIC** | **Weight 1** | **Weight 2** |
| --- | --- | --- | --- | --- | --- | --- | --- | --- | --- | --- |
| 20 | + | + | + | p+m+p:m | 8 | -1056.5 | 2129.4 | 0 | 0.86 | 0.86 |
| 16 | + | + |  | p+m+p:m | 7 | -1060 | 2134.2 | 4.8 | 0.08 | 0.94 |
| 12 |  | + |  | p+m+p:m | 6 | -1061.3 | 2134.8 | 5.4 | 0.06 | 1.00 |
| 08 | + |  |  | p+m+p:m | 6 | -1064.1 | 2140.4 | 10.9 | 0 | 1.00 |
| 04 |  |  |  | p+m+p:m | 5 | -1065.7 | 2141.5 | 12.1 | 0 | 1.00 |
| 18 | + | + | + | p | 6 | -1074.6 | 2161.4 | 32 | 0 | 1.00 |
| 19 | + | + | + | p+m | 7 | -1074.5 | 2163.2 | 33.8 | 0 | 1.00 |
| 14 | + | + |  | p | 5 | -1077.7 | 2165.6 | 36.2 | 0 | 1.00 |
| 10 |  | + |  | p | 4 | -1079.3 | 2166.7 | 37.3 | 0 | 1.00 |
| 15 | + | + |  | p+m | 6 | -1077.5 | 2167.2 | 37.8 | 0 | 1.00 |
| 11 |  | + |  | p+m | 5 | -1079.1 | 2168.3 | 38.9 | 0 | 1.00 |
| 17 | + | + | + | m | 6 | -1079.3 | 2170.9 | 41.4 | 0 | 1.00 |
| 13 | + | + |  | m | 5 | -1082.1 | 2174.3 | 44.9 | 0 | 1.00 |
| 07 | + |  |  | p+m | 5 | -1082.5 | 2175.1 | 45.7 | 0 | 1.00 |
| 03 |  |  |  | p+m | 4 | -1084.5 | 2177 | 47.6 | 0 | 1.00 |
| 05 | + |  |  | m | 4 | -1086.7 | 2181.4 | 52 | 0 | 1.00 |
| 09 |  | + |  | m | 4 | -1087.5 | 2183.1 | 53.7 | 0 | 1.00 |
| 06 | + |  |  | p | 4 | -1091.8 | 2191.6 | 62.2 | 0 | 1.00 |
| 02 |  |  |  | p | 3 | -1094 | 2194.1 | 64.7 | 0 | 1.00 |
| 01 |  |  |  | m | 3 | -1099.1 | 2204.2 | 74.8 | 0 | 1.00 |

**Table S7. Model-averaged parameter estimates are shown for the log-transformed *Bd* zoospore load of the 2010 cohort of *A*. *obstetricans* tadpoles.** Shown are the parameters types, the parameters names, the parameter estimates, and the 95% confidence limits (LL = lower limit and UL = upper limit). Estimate 1 is averaged over all the models in the set. Estimate 2 is averaged over the subset of models with a cumulative support of 95%. The 95% confidence limits are for estimate 2.

| **Type** | **Name** | **Estimate 1** | **Estimate 2** | **95% LL** | **95% UL** |
| --- | --- | --- | --- | --- | --- |
| Intercept^a^ | Control ponds | 1.93115 | 1.93115 | 1.041860 | 2.820441 |
| Contrast1^b^ | Fungicide ponds | 0.7943654 | 0.845296 | -0.367326 | 2.057917 |
| Slope^c^ | Time | -9.438511 | -9.491714 | -26.268115 | 7.284686 |
| Contrast2^d^ | Fungicide:Time | -1.853242 | -2.161358 | -26.361237 | 22.038521 |

^a^ Value of the intercept of the control ponds on May 5, 2011

^b^ Value of the contrast1[intercept(fungicide) – intercept(control)] on May 5, 2011

^c^ Value of the slope of log10(*Bd* zoospore load) versus time in the control ponds

^b^ Value of the contrast2[slope(fungicide) – slope(control)]

**Table S8. Model selection results are shown for the log10-transformed *Bd* zoospore load of the 2011 cohort of *A. obstetricans* tadpoles.** The log10-transformed *Bd* zoospore load was modelled as a linear mixed effects model with normal errors. Fixed factors included fungicide treatment (F), the linear term of time (T), the quadratic term of time (T^2^), and their interactions (F:T; F:T^2^). Random factors included pond (p), month (m), and their interaction (p:m). Shown for each model are: the model ID (ID), the fixed effects structure (F, T, T^2^, F:T, and F:T^2^), the random effects structure (Random), the model degrees of freedom (Df), the log-likelihood (logLik), the Akaike information criterion (AIC), the difference in the AIC value from the top model (Δ AIC), the model weight (weight 1), and cumulative weight (weight 2).

| **ID** | **F** | **T** | **T^2^** | **F:T** | **F:T^2^** | **Random** | **Df** | **logLik** | **AIC** | **Δ AIC** | **Weight 1** | **Weight 2** |
| --- | --- | --- | --- | --- | --- | --- | --- | --- | --- | --- | --- | --- |
| 36 | + | + | + | + | + | p+m+p:m | 10 | -2375.9 | 4772.1 | 0.00 | 0.855 | 0.86 |
| 32 | + | + | + | + |  | p+m+p:m | 9 | -2378.8 | 4775.8 | 3.77 | 0.130 | 0.99 |
| 28 | + | + | + |  |  | p+m+p:m | 8 | -2382.1 | 4780.4 | 8.29 | 0.014 | 1.00 |
| 16 |  | + | + |  |  | p+m+p:m | 7 | -2385.0 | 4784.1 | 12.02 | 0.00 | 1.00 |
| 24 | + | + |  | + |  | p+m+p:m | 8 | -2387.7 | 4791.5 | 19.47 | 0.00 | 1.00 |
| 20 | + | + |  |  |  | p+m+p:m | 7 | -2391.0 | 4796.2 | 24.12 | 0.00 | 1.00 |
| 12 |  | + |  |  |  | p+m+p:m | 6 | -2393.9 | 4799.9 | 27.82 | 0.00 | 1.00 |
| 08 | + |  |  |  |  | p+m+p:m | 6 | -2395.0 | 4802.0 | 29.92 | 0.00 | 1.00 |
| 04 |  |  |  |  |  | p+m+p:m | 5 | -2397.8 | 4805.7 | 33.63 | 0.00 | 1.00 |
| 35 | + | + | + | + | + | p+m | 9 | -2399.4 | 4816.9 | 44.85 | 0.00 | 1.00 |
| 31 | + | + | + | + |  | p+m | 8 | -2401.8 | 4819.7 | 47.59 | 0.00 | 1.00 |
| 27 | + | + | + |  |  | p+m | 7 | -2404.9 | 4823.9 | 51.81 | 0.00 | 1.00 |
| 15 |  | + | + |  |  | p+m | 6 | -2407.3 | 4826.8 | 54.70 | 0.00 | 1.00 |
| 23 | + | + |  | + |  | p+m | 7 | -2411.4 | 4836.9 | 64.81 | 0.00 | 1.00 |
| 33 | + | + | + | + | + | m | 8 | -2410.9 | 4837.9 | 65.81 | 0.00 | 1.00 |
| 34 | + | + | + | + | + | p | 8 | -2412.2 | 4840.5 | 68.39 | 0.00 | 1.00 |
| 29 | + | + | + | + |  | m | 7 | -2413.2 | 4840.5 | 68.41 | 0.00 | 1.00 |
| 19 | + | + |  |  |  | p+m | 6 | -2414.7 | 4841.4 | 69.32 | 0.00 | 1.00 |
| 30 | + | + | + | + |  | p | 7 | -2414.5 | 4843.0 | 70.98 | 0.00 | 1.00 |
| 25 | + | + | + |  |  | m | 6 | -2416.1 | 4844.3 | 72.19 | 0.00 | 1.00 |
| 11 |  | + |  |  |  | p+m | 5 | -2417.1 | 4844.3 | 72.20 | 0.00 | 1.00 |
| 26 | + | + | + |  |  | p | 6 | -2417.2 | 4846.4 | 74.35 | 0.00 | 1.00 |
| 07 | + |  |  |  |  | p+m | 5 | -2418.6 | 4847.2 | 75.12 | 0.00 | 1.00 |
| 14 |  | + | + |  |  | p | 5 | -2419.4 | 4848.8 | 76.74 | 0.00 | 1.00 |
| 03 |  |  |  |  |  | p+m | 4 | -2421.0 | 4850.1 | 78.02 | 0.00 | 1.00 |
| 21 | + | + |  | + |  | m | 6 | -2422.7 | 4857.4 | 85.38 | 0.00 | 1.00 |
| 17 | + | + |  |  |  | m | 5 | -2425.7 | 4861.5 | 89.41 | 0.00 | 1.00 |
| 05 | + |  |  |  |  | m | 4 | -2429.6 | 4867.2 | 95.16 | 0.00 | 1.00 |
| 13 |  | + | + |  |  | m | 5 | -2445.6 | 4901.2 | 129.16 | 0.00 | 1.00 |
| 09 |  | + |  |  |  | m | 4 | -2455.0 | 4917.9 | 145.88 | 0.00 | 1.00 |
| 01 |  |  |  |  |  | m | 3 | -2458.9 | 4923.8 | 151.72 | 0.00 | 1.00 |
| 22 | + | + |  | + |  | l | 6 | -2500.0 | 5012.1 | 240.00 | 0.00 | 1.00 |
| 18 | + | + |  |  |  | l | 5 | -2503.0 | 5016.1 | 244.00 | 0.00 | 1.00 |
| 06 | + |  |  |  |  | l | 4 | -2504.7 | 5017.4 | 245.34 | 0.00 | 1.00 |
| 10 |  | + |  |  |  | l | 4 | -2505.2 | 5018.4 | 246.35 | 0.00 | 1.00 |
| 02 |  |  |  |  |  | l | 3 | -2506.9 | 5019.7 | 247.68 | 0.00 | 1.00 |

**Table S9. Model-averaged parameter estimates are shown for the log-transformed *Bd* zoospore load of the 2011 cohort of *A*. *obstetricans* tadpoles.** Shown are the parameters types, the parameters names, the parameter estimates, and the 95% confidence limits (LL = lower limit and UL = upper limit). Estimate 1 is averaged over all the models in the set. Estimate 2 is averaged over the subset of models with a cumulative support of 95%. The 95% confidence limits are for estimate 2.

| **Type** | **Name** | **Estimate 1** | **Estimate 2** | **95% LL** | **95% UL** |
| --- | --- | --- | --- | --- | --- |
| Intercept^a^ | Control ponds | 3.186082 | 3.186082 | 2.744230 | 3.627934 |
| Contrast1^b^ | Fungicide ponds | 0.922980 | 0.924919 | 0.399994 | 1.449844 |
| Slope1^c^ | Time | -1.73608 | -1.73608 | -14.53335 | 11.061189 |
| Contrast2^d^ | Fungicide:Time | 5.989391 | 6.084410 | -8.711603 | 20.880423 |
| Slope2^e^ | Time^2^g^ | 27.86999 | 27.87157 | 16.069282 | 39.673867 |
| Contrast3^f^ | Fungicide:Time^2 | 1.045272 | 1.222885 | -12.97272 | 15.418485 |

^a^ Logit value of the intercept of the control ponds on December 27, 2011

^b^ Logit value of the contrast1[intercept(fungicide) – intercept(control)] on Dec 27, 2011

^c^ Logit value of the slope1 of log10(*Bd* zoospore load) versus time in the control ponds

^d^ Logit value of the contrast2[slope1(fungicide) – slope1(control)]

^e^ Logit value of the slope2 of log10(*Bd* zoospore load) versus time^2 in the control ponds

^f^ Logit value of the contrast3[slope2(fungicide) – slope2(control)]

^g^ The transformed time^2 variable is a negative quadratic function. A positive slope2 means that the *Bd* prevalence is highest in the winter and lower in the fall and spring

**Table S10. Model selection results are shown for the effect of temperature on *Bd* prevalence of the 2011 cohort of *A. obstetricans* tadpoles.** The *Bd* prevalence was modeled as a generalized linear mixed effects model with binomial errors. Fixed factors included the linear term of temperature (C), the quadratic term of temperature (C^2^), the linear term of time (T), the quadratic term of time (T^2^). Random factors included pond (p), month (m), and their interaction (p:m). Shown for each model are: the model ID (ID), the fixed effects structure (C, C^2^, T, T^2^), the random effects structure (Random), the model degrees of freedom (Df), the log-likelihood (logLik), the Akaike information criterion (AIC), the difference in the AIC value from the top model (Δ AIC), the model weight (Weight 1), and the cumulative weight (Weight 2).

| **ID** | **C** | **C^2^** | **T** | **T^2^** | **Random** | **Df** | **logLik** | **AIC** | **Δ AIC** | **Weight 1** | **Weight 2** |
| --- | --- | --- | --- | --- | --- | --- | --- | --- | --- | --- | --- |
| 32 | + |  | + | + | p+m+p:m | 7 | -518.6 | 1051.3 | 0.00 | 0.374 | 0.37 |
| 20 |  |  | + | + | p+m+p:m | 6 | -519.9 | 1051.8 | 0.49 | 0.293 | 0.67 |
| 12 |  |  | + |  | p+m+p:m | 5 | -521.6 | 1053.3 | 2.05 | 0.134 | 0.80 |
| 24 | + |  | + |  | p+m+p:m | 6 | -521.6 | 1055.3 | 4.02 | 0.050 | 0.85 |
| 04 |  |  |  |  | p+m+p:m | 4 | -523.7 | 1055.3 | 4.06 | 0.049 | 0.90 |
| 08 | + |  |  |  | p+m+p:m | 5 | -522.8 | 1055.7 | 4.46 | 0.040 | 0.94 |
| 16 | + | + |  |  | p+m+p:m | 6 | -522.0 | 1056.1 | 4.87 | 0.033 | 0.97 |
| 28 | + | + | + |  | p+m+p:m | 7 | -521.2 | 1056.5 | 5.26 | 0.027 | 1.00 |
| 31 | + |  | + | + | p+m | 6 | -537.9 | 1087.9 | 36.61 | 0.000 | 1.00 |
| 23 | + |  | + |  | p+m | 5 | -542.4 | 1094.8 | 43.50 | 0.000 | 1.00 |
| 27 | + | + | + |  | p+m | 6 | -541.7 | 1095.5 | 44.19 | 0.000 | 1.00 |
| 07 | + |  |  |  | p+m | 4 | -545.6 | 1099.2 | 47.87 | 0.000 | 1.00 |
| 15 | + | + |  |  | p+m | 5 | -544.6 | 1099.2 | 47.97 | 0.000 | 1.00 |
| 19 |  |  | + | + | p+m | 5 | -546.1 | 1102.3 | 51.03 | 0.000 | 1.00 |
| 11 |  |  | + |  | p+m | 4 | -547.8 | 1103.6 | 52.37 | 0.000 | 1.00 |
| 03 |  |  |  |  | p+m | 3 | -550.5 | 1106.9 | 55..66 | 0.000 | 1.00 |
| 29 | + |  | + | + | m | 5 | -559.0 | 1128.0 | 76.74 | 0.000 | 1.00 |
| 25 | + | + | + |  | m | 5 | -560.0 | 1130.0 | 78.72 | 0.000 | 1.00 |
| 13 | + | + |  |  | m | 4 | -563.3 | 1134.7 | 83.38 | 0.000 | 1.00 |
| 21 | + |  | + |  | m | 4 | -564.1 | 1136.3 | 84.99 | 0.000 | 1.00 |
| 05 | + |  |  |  | m | 3 | -567.9 | 1141.8 | 90.50 | 0.000 | 1.00 |
| 17 |  |  | + | + | m | 4 | -573.3 | 1154.6 | 103.29 | 0.000 | 1.00 |
| 09 |  |  | + |  | m | 3 | -575.3 | 1156.5 | 105.26 | 0.000 | 1.00 |
| 01 |  |  |  |  | m | 2 | -578.1 | 1160.2 | 108.91 | 0.000 | 1.00 |
| 18 |  |  | + | + | p | 4 | -580.1 | 1168.2 | 116.88 | 0.000 | 1.00 |
| 30 | + |  | + | + | p | 5 | -580.0 | 1170.1 | 118.80 | 0.000 | 1.00 |
| 26 | + | + | + |  | p | 5 | -582.1 | 1174.2 | 122.87 | 0.000 | 1.00 |
| 14 | + | + |  |  | p | 4 | -585.8 | 1179.6 | 128.34 | 0.000 | 1.00 |
| 22 | + |  | + |  | p | 4 | -588.0 | 1184.1 | 132.78 | 0.000 | 1.00 |
| 06 | + |  |  |  | p | 3 | -601.3 | 1208.6 | 157.36 | 0.000 | 1.00 |
| 10 |  |  | + |  | p | 3 | -602.8 | 1211.7 | 160.37 | 0.000 | 1.00 |
| 02 |  |  |  |  | p | 2 | -667.8 | 1339.7 | 288.40 | 0.000 | 1.00 |

**Table S11. Model-averaged parameter estimates are shown for the effect of temperature on *Bd* prevalence of the 2011 cohort of *A*. *obstetricans* tadpoles.** Shown are the parameters types, the parameters names, the parameter estimates, and the 95% confidence limits (LL = lower limit and UL = upper limit). Estimate 1 is averaged over all the models in the set. Estimate 2 is averaged over the subset of models with a cumulative support of 95%. The 95% confidence limits are for estimate 2.

| **Type** | **Name** | **Estimate 1** | **Estimate 2** | **95% LL** | **95% UL** |
| --- | --- | --- | --- | --- | --- |
| Intercept^a^ | Intercept | 2.221086 | 2.221086 | 1.001506 | 3.440665 |
| Slope1^b^ | Temperature | 17.38547 | 33.18063 | -48.974494 | 115.335758 |
| Slope2^c^ | Temperature^2^f^ | -0.741487 | -12.414459 | -34.812962 | 9.984045 |
| Slope3^d^ | Time | 42.72144 | 48.67187 | 1.054406 | 96.289343 |
| Slope4^e^ | Time^2^g^ | 33.17957 | 49.77178 | -2.835467 | 102.379026 |

^a^ Logit value of the intercept of the ponds on December 16, 2011; ~4.34°C

^b^ Logit value of the slope1 of *Bd* prevalence versus temperature

^c^ Logit value of the slope2 of *Bd* prevalence versus temperature^2

^d^ Logit value of the slope3 of *Bd* prevalence versus time

^e^ Logit value of the slope4 of *Bd* prevalence versus time^2

^f^ The transformed temperature^2 variable is a positive quadratic function. A negative slope2 means that the *Bd* prevalence is highest in the winter and lower in the fall and spring

^g^ The transformed time^2 variable is a negative quadratic function. A positive slope4 means that the *Bd* prevalence is highest in the winter and lower in the fall and spring

Range of Time (before dividing by 30): 1 to 246 days

Mean of Time (before dividing by 30): 136.9 days (December 16, 2011; ~4.34°C)

Zero point for Time: 136.9 days (December 16, 2011; ~4.34°C)

Vertex for Time^2^: 136.9 days (December 16, 2011; ~4.34°C)

Range of Temperature: 2.16 to 18.21 °C

Mean of Temperature: 9.48°C (~November 4, 2011)

Zero point for Temperature: 9.48°C (~November 4, 2011)

Vertex for Temperature^2^ 9.48°C (~November 4, 2011)

**Table S12. Model selection results are shown for the effect of temperature on log-transformed *Bd* zoospore load of the 2011 cohort of *A. obstetricans* tadpoles.** The log-transformed *Bd* zoospore load was modeled as a linear mixed effects model with normal errors. Fixed factors included the linear term of temperature (C), the quadratic term of temperature (C^2^), the linear term of time (T), the quadratic term of time (T^2^). Random factors included pond (p), month (m), and their interaction (p:m). Shown for each model are: the model ID (ID), the fixed effects structure (C, C^2^, T, T^2^), the random effects structure (Random), the model degrees of freedom (Df), the log-likelihood (logLik), the Akaike information criterion (AIC), the difference in the AIC value from the top model (Δ AIC), the model weight (Weight 1), and the cumulative weight (Weight 2).

| **ID** | **C** | **C^2^** | **T** | **T^2^** | **Random** | **Df** | **logLik** | **AIC** | **Δ AIC** | **Weight 1** | **Weight 2** |
| --- | --- | --- | --- | --- | --- | --- | --- | --- | --- | --- | --- |
| 32 | + |  | + | + | p+m+p:m | 8 | -1999.3 | 4014.8 | 0.00 | 0.987 | 0.99 |
| 20 |  |  | + | + | p+m+p:m | 7 | -2004.7 | 4023.6 | 8.78 | 0.012 | 1.00 |
| 28 | + | + | + |  | p+m+p:m | 8 | -2006.5 | 4029.1 | 14.32 | 0.000 | 1.00 |
| 24 | + |  | + |  | p+m+p:m | 7 | -2009.4 | 4032.8 | 18.03 | 0.000 | 1.00 |
| 16 | + | + |  |  | p+m+p:m | 7 | -2009.9 | 4034.0 | 19.17 | 0.000 | 1.00 |
| 31 | + |  | + | + | p+m | 7 | -2010.0 | 4034.1 | 19.33 | 0.000 | 1.00 |
| 12 |  |  | + |  | p+m+p:m | 6 | -2012.7 | 4037.4 | 22.62 | 0.000 | 1.00 |
| 08 | + |  |  |  | p+m+p:m | 6 | -2012.9 | 4037.9 | 23.14 | 0.000 | 1.00 |
| 04 |  |  |  |  | p+m+p:m | 5 | -2016.5 | 4043.0 | 28.23 | 0.000 | 1.00 |
| 27 | + | + | + |  | p+m | 7 | -2019.4 | 4053.0 | 38.21 | 0.000 | 1.00 |
| 23 | + |  | + |  | p+m | 6 | -2021.8 | 4055.7 | 4089 | 0.000 | 1.00 |
| 19 |  |  | + | + | p+m | 6 | -2021.8 | 4055.7 | 40.90 | 0.000 | 1.00 |
| 30 | + |  | + | + | p | 6 | -2025.0 | 4062.0 | 47.24 | 0.000 | 1.00 |
| 15 | + | + |  |  | p+m | 6 | -2025.2 | 4062.5 | 47.66 | 0.000 | 1.00 |
| 07 | + |  |  |  | p+m | 5 | -2027.6 | 4065.3 | 50.49 | 0.000 | 1.00 |
| 11 |  |  | + |  | p+m | 5 | -2030.3 | 4070.7 | 55.88 | 0.000 | 1.00 |
| 18 |  |  | + | + | p | 5 | -2032.4 | 4074.8 | 60.03 | 0.000 | 1.00 |
| 03 |  |  |  |  | p+m | 4 | -2034.0 | 4076.1 | 61.26 | 0.000 | 1.00 |
| 29 | + |  | + | + | m | 6 | -2035.6 | 4083.2 | 68.43 | 0.000 | 1.00 |
| 25 | + | + | + |  | m | 6 | -2041.5 | 4095.1 | 80.36 | 0.000 | 1.00 |
| 13 | + | + |  |  | m | 5 | -2046.1 | 4102.3 | 87.56 | 0.000 | 1.00 |
| 21 | + |  | + |  | m | 5 | -2046.4 | 4102.8 | 87.98 | 0.000 | 1.00 |
| 26 | + | + | + |  | p | 6 | -2046.5 | 4105.1 | 90.28 | 0.000 | 1.00 |
| 17 |  |  | + | + | m | 5 | -2048.8 | 4107.6 | 92.77 | 0.000 | 1.00 |
| 05 | + |  |  |  | m | 4 | -2051.6 | 4111.2 | 96.45 | 0.000 | 1.00 |
| 22 | + |  | + |  | p | 5 | -2054.1 | 4118.3 | 103.50 | 0.000 | 1.00 |
| 09 |  |  | + |  | m | 4 | -2057.1 | 4122.2 | 107.41 | 0.000 | 1.00 |
| 01 |  |  |  |  | m | 3 | -2060.8 | 4127.7 | 112.87 | 0.000 | 1.00 |
| 14 | + | + |  |  | p | 5 | -2060.6 | 4131.3 | 116.49 | 0.000 | 1.00 |
| 06 | + |  |  |  | p | 4 | -2062.7 | 4133.5 | 118.68 | 0.000 | 1.00 |
| 10 |  |  | + |  | p | 4 | -2088.4 | 4184.9 | 170.12 | 0.000 | 1.00 |
| 02 |  |  |  |  | p | 3 | -2090.2 | 4186.4 | 171.58 | 0.000 | 1.00 |

**Table S13. Model-averaged parameter estimates are shown for the effect of temperature on log-transformed *Bd* zoospore load of the 2011 cohort of *A*. *obstetricans* tadpoles.** Shown are the parameters types, the parameters names, the parameter estimates, and the 95% confidence limits (LL = lower limit and UL = upper limit). Estimate 1 is averaged over all the models in the set. Estimate 2 is averaged over the subset of models with a cumulative support of 95%. The 95% confidence limits are for estimate 2.

| **Type** | **Name** | **Estimate 1** | **Estimate 2** | **95% LL** | **95% UL** |
| --- | --- | --- | --- | --- | --- |
| Intercept^a^ | Intercept | 3.805913 | 3.805913 | 3.238466 | 4.373360 |
| Slope1^b^ | Temperature | 23.52743 | 23.81931 | 2.047364 | 45.591254 |
| Slope2^c^ | Temperature^2^f^ | -0.002568 | -3.079942 | -15.752306 | 9.592422 |
| Slope2^d^ | Time | 10.52030 | 10.52112 | -3.906527 | 24.948770 |
| Slope4^e^ | Time^2^g^ | 39.59317 | 39.63184 | 20.940295 | 58.323391 |

^a^ Value of the intercept of the ponds on December 29, 2011; ~4.08°C

^b^ Value of the slope1 of log10(*Bd* zoospore load) versus temperature

^c^ Value of the slope2 of log10(*Bd* zoospore load) versus temperature^2

^d^ Value of the slope3 of log10(*Bd* zoospore load) versus time

^e^ Value of the slope4 of log10(*Bd* zoospore load) versus time^2

^f^ The transformed temperature^2 variable is a positive quadratic function. A negative slope2 means that the *Bd* zoospore load is highest in the winter and lower in the fall and spring

^g^ The transformed time^2 variable is a negative quadratic function. A positive slope4 means that the *Bd* zoospore load is highest in the winter and lower in the fall and spring

Range of Time (before dividing by 30): 1 to 246 days

Mean of Time (before dividing by 30): 150.5 days (December 29, 2011; ~4.08°C)

Zero point for Time: 150.5 days (December 29, 2011; ~4.08°C)

Vertex for Time^2^: 150.5 days (December 29, 2011; ~4.08°C)

Range of Temperature: 2.16 to 18.21 °C

Mean of Temperature: 8.75 °C (~November 10, 2011)

Zero point for Temperature: 8.75 °C (~November 10, 2011)

Vertex for Temperature^2^: 8.75 °C (~November 10, 2011)


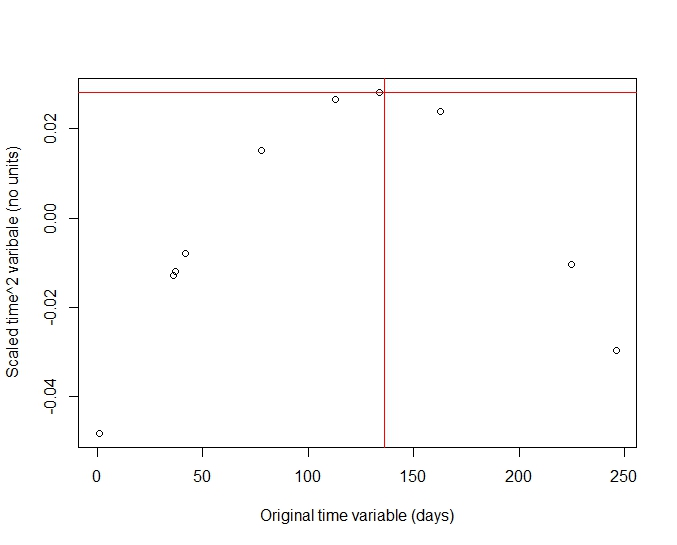


Figure S1. The scaled time^2 variable (no units) is a negative quadratic function of the original time variable (days). The original time^2 variable (days^2) was scaled using the poly() function in R. The vertex of the rescaled variable occurs at day 137 on the original time scale (y = 0.03046672). The time data were taken from the dataset that contained all the tadpoles of the 2011 cohort (n = 1164 tadpoles).


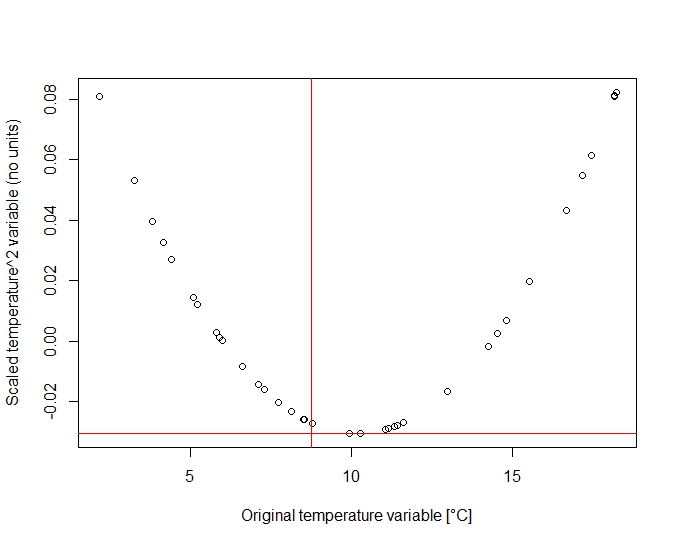


Figure S2. The scaled temperature^2 variable (no units) is a positive quadratic function of the original temperature variable (°C). The original temperature^2 variable (°C^2) was scaled using the poly() function in R. The vertex of the rescaled variable occurs at a temperature of 9.48°C on the original time scale (y = -0.02940708). The temperature data were taken from the dataset that contained all the tadpoles of the 2011 cohort (n = 967 tadpoles) and excluded the SchU pond because the temperature logger failed.
